# Evidence for Two Soybean Looper Strains in the United States with Limited Capacity for Cross-Hybridization

**DOI:** 10.3390/genes14071509

**Published:** 2023-07-24

**Authors:** Rodney N. Nagoshi, Jeffrey A. Davis, Robert L. Meagher, Fred R. Musser, Graham P. Head, Hector Portillo, Henry Teran

**Affiliations:** 1 Center for Medical, Agricultural and Veterinary Entomology, United States Department of Agriculture, Agricultural Research Service, Gainesville, FL 32608, USA; rob.meagher@usda.gov; 2 Department of Entomology, LSU Agricultural Center, 404 Life Science Building, Baton Rouge, LA 70803, USA; jeffdavis@agcenter.lsu.edu; 3 Department of Biochemistry, Molecular Biology, Entomology, and Plant Pathology, Mississippi State University, Starkville, MS 39762, USA; fmusser@entomology.msstate.edu; 4 Bayer Crop Science US, Chesterfield, MO 63017, USA; graham.head@bayer.com; 5 FMC Agricultural Solutions, Stine Research Center, Newark, DE 19711, USA; hector.portillo@fmc.com; 6 Corteva Agriscience™, Carr #3 Km 156.5, Salinas, PR 00751, USA; henry.teransantofimio@corteva.com

**Keywords:** soybean looper, migration, haplotypes, fall armyworm, strains, genetic structure

## Abstract

The noctuid moth soybean looper (SBL), *Chrysodeixis includens* (Walker) is an economically important pest of soybeans (*Glycine max* (L.) Merr.) in the southeastern United States. It has characteristics that are of particular concern for pest mitigation that include a broad host range, the capacity for annual long-distance flight, and resistance in some populations to important pesticides such as pyrethroids and chitin synthesis inhibitor. The biology of SBL in the United States resembles that of the fellow noctuid fall armyworm (FAW), *Spodoptera frugiperda* (J.E. Smith), a major pest of corn and several other crops. FAW exhibits a population structure in that it can be divided into two groups (host strains) that differ in their host preferences but are broadly sympatric and exhibit incomplete reproductive isolation. In this paper, strategies used to characterize the FAW strains were applied to SBL to assess the likelihood of population structure in the United States. Evidence is presented for two SBL strains that were defined phylogenetically and display differences in the proportions of a small set of genetic markers. The populations exhibit evidence of reproductive barriers sufficient to allow persistent asymmetry in the distribution of mitochondrial haplotypes. The identified molecular markers will facilitate studies characterizing the behaviors of these two populations, with relevance to pest mitigation and efforts to prevent further dispersal of the resistance traits.

## 1. Introduction

Phytophagous insects can be classified into three categories based on feeding behavior: monophagous insects feed on a single plant species, oligophagous insects feed on several species within closely related families, and polyphagous insects have the ability to feed across higher phylogenetic groupings. Polyphagous agricultural pests capable of using a multiplicity of plant species present added challenges for mitigation strategies that include an increased number of habitats that can support pest populations. This makes extrapolating the source of migratory populations more difficult and complicates projections of future infestations. Fortunately, a more extreme phytophagy is relatively rare, perhaps because of unfavorable competition with more specialized, better-adapted species [1]. This would restrict the host range over time, with one possible outcome being a form of oligophagy where a few plant species are adapted to as primary hosts and are preferred by the pest, while it retains some capacity to use a wider range of secondary host species [2].

The soybean looper (SBL), *Chrysodeixis includens* (Walker), and fall armyworm (FAW), *Spodoptera frugiperda* (J.E. Smith), are noctuid moth species that appear to exhibit this type of oligophagous feeding behavior. SBL has a broad host range that includes over 70 plant species but exhibits the most economic damage as a defoliator of soybean, while also causing significant yield losses in cotton, bean, and tomato crops [3]. FAW has a similarly broad and partially overlapping host range that is in part due to the existence of two populations that differ in host usage [4,5]. The C-strain is preferentially found on corn and sorghum while the R-strain predominates in millet and pasture grasses [6,7]. This may be an example of speciation coincident with substantial gene flow similar to that described in the Lepidoptera species complex *Heliconius*, where divergence in some parts of the genome occurred contemporaneously with persistent admixture [8].

There is also evidence for at least two genetically distinct populations of SBL al-though the data are more suggestive than conclusive. Collections in Brazil were characterized via inter-simple sequence repeat (ISSR) analysis, in which geographical populations were compared in terms of microsatellite-based polymorphisms [9]. Principal coordinate analysis revealed low but significant genetic differentiation between two groups that were sympatric, showed no geographical specificity, and were associated with a high gene flow and low genetic diversity. It was suggested that the two ISSR groups may be the result of a recent recolonization of SBL into Brazil [9]. This would presumably be a transient differentiation that will disappear as the populations continue to cross-hybridize. A subsequent study of Brazilian SBL using a combination of mitochondrial haplotypes and nuclear SNPs came to similar conclusions of overall low genetic diversity with little genetic structure [10]. However, genetic differences were detected between specimens collected from soybean and cotton in one location, suggesting a possible host-driven divergence of populations, but this difference was not observed in collections from a second location. A limitation of both studies is that sampling was from a single year, so the persistence of the observations is not known.

In this study, we tested for genetically differentiated groups in the North American SBL population. This was carried out by applying a methodology developed and successfully used to detect and characterize the FAW strains in wild populations. The strategy is based on two premises. The first is that while moderate gene flow resulting from incomplete reproductive isolation may not allow population-specific mitochondrial haplotypes, differences in the relative proportions of haplotypes might form and be detectable. The second is that the Z-chromosome may play a disproportionate role in speciation [11,12] with supporting evidence documented in several Lepidoptera species [13,14] including FAW [15]. Particularly relevant to this study are reports that polymorphisms in the Z-linked *Triosephosphate isomerase* (*Tpi*) gene can differentiate closely related moth populations such as the FAW host strains [16], the sibling *Helicoverpa* species of *H. armigera* and *H. zea* [17], and the *E* and *Z* pheromone strains of *Ostrinia nubilalis* [18]. The *Tpi* product is a housekeeping function and so is unlikely to drive population divergence, leaving its physical linkage to other Z-chromosome functions as the most likely explanation for its effectiveness as a genetic marker.

Based on these observations, SBL specimens were collected from multiple locations in the U.S. over multiple years and characterized for a segment of the mitochondrial *Cytochrome oxidase subunit I* (*COI*) gene previously used to differentiate geographical FAW populations [19], and a highly variable intron segment from the SBL *Tpi* gene, the analog of which in FAW produced a phylogenetic description of the two host strains [17,20]. Evidence was found for two SBL populations with restricted capacity to cross-hybridize.

## 2. Materials and Methods

### 2.1. Specimen Collections

Specimens were collected from soybean in field plots either through pheromone trapping (adult males) or as larvae picked directly from soybean (Table 1A). In addition, larvae collected from soybean were reared to produce laboratory colonies, and specimens from these collections were obtained after the first (F1) or second (F2) generation (Table 1B). The collected specimens were stored in ethanol and initially identified as SBL according to morphological criteria before molecular analysis. The gender of the larval and colony specimens was not identified.

### 2.2. DNA Preparation

A mix of genomic and mitochondrial DNA from individual specimens was purified from individuals as described in [21], and stored in TE (10 mM Tris (pH 8), and 1 mM EDTA) at −20 °C. The procedure involved using Genomic Lysis Buffer and Zymo-Spin III columns (Zymo Research, Orange, CA, USA), following the manufacturer’s instructions. DNA preparation was increased to a final volume of 100 µL with distilled water. Genomic DNA preparations were stored at −20 °C and analyzed as needed.

### 2.3. Isolation of the DNA Segments by PCR

Segments of the SBL *COI* gene sequence were identified by aligning the well-characterized FAW *COI* sequences with the SBL mitochondria genome sequence (GenBank LR797832). The primers used to amplify the sCOIB segment were sC891f (5′-TACACGAGCTTATTTTACTTC-3′), and sC1457r (5′-ATATCATTCAATAGAAGAGG-3′). PCR amplification was performed in a 30 µL reaction mix containing 3 µL of 10× manufacturer’s reaction buffer, 1 µL of 10 mM dNTP, 0.5 µL of a 20 µM primer mix, 1 µL of the DNA template (between 0.05–0.5 µg), and 0.5 units of Taq DNA polymerase (New England Biolabs, Beverly, MA, USA). The thermocycling program was 94 °C (1 min), followed by 33 cycles of 92 °C (30 s), 56 °C (30 s), 72 °C (30 s), and a final segment of 72 °C for 3 min. Briefly, 96 PCR amplifications were performed at the same time using either 0.2 mL tube strips or 96 well-microtiter plates. 

The sTpiI140 segment was amplified by the primer pair sTpi891f/sTpi1140r using the same protocol as that used for sCOIB. All primers used for PCR and DNA sequencing were synthesized by Integrated DNA Technologies (Coralville, IA, USA). These included sC891f (5′-TACACGAGCTTATTTTACTTC-3′), sC1457r (5′-ATATCATTCAATAGAAGAGG-3′), sTpi412f (5′-ATGGCCTGAAAGTCATTGCCTG-3′), and sTpi1140r (5′-GCAGACACATTCTTAGCCAGCC-3′).

For fragment isolations, 6 µL of 6× gel loading buffer was added to the amplification reaction and the entire sample run on 1.8% agarose horizontal gel containing GelGreen (Biotium, Hayward, CA, USA) in 0.5× Tris-borate buffer (TBE, 45 mM Tris base, 45 mM boric acid, and 1 mM EDTA; pH 8.0). Fragments were visualized on a Blue LED transilluminator (Thermo Fisher Scientific, Waltham, MA, USA) and cut out from the gel. Fragment isolation was performed using Zymo-Spin I columns (Zymo Research, Orange, CA, USA) in accordance with the manufacturer’s instructions. 

### 2.4. Characterization of Haplotypes

Each specimen was anticipated to have a single *COI* haplotype that was maternally inherited. The sCOIB haplotype classes used in this study are defined by SNPs sC1035 and sC1272 that are each polymorphic for C/T and produce four observed combinations (C_1035_C_1272_, C_1035_T_1272_, T_1035_C_1272_, and T_1035_T_1272_), with C_1035_T_1272_ and T_1035_C_1272_ being the predominant forms. To simplify the analysis, the sCOIB mitochondrial haplotype categories in this paper were defined solely by sC1035 polymorphisms and designated as either C1035_COI_ or T1035_COI_ if a C or T was present, respectively.

The SBL *Tpi* hapotypes are more complicated as the location of the *Tpi* gene on the Z-chromosome means that two gene copies are present in males (*Z*/*Z*) and so heterozygous allele combinations are possible, while females (*Z*/*W*) carry only a single copy. Because DNA sequencing is performed on PCR fragments directly produced from the specimen, heterozygosity for the *Tpi* segment will result in the simultaneous sequencing of two different alleles. This will produce chromatographs with overlapping curves at the sequence mismatch. Particularly disruptive are heterozygous indels (insertions and deletions) because these can shift the DNA sequencing frame of the two DNA strands, resulting in continued mismatch downstream of the indel. 

### 2.5. DNA Sequence Analysis, Statistics, and Data Availability

The isolated fragments were analyzed via DNA sequencing using the appropriate primers (Azenta, South Plainfield, NJ, USA). DNA comparisons and alignments were performed using the Geneious Pro 10.1.2 (https://www.geneious.com, accessed on 15 June 2023) [22]. All sCOIB haplotypes described in this study were deposited in GenBank, (accession nos. OR128567-OR128591) as were the collection of sTpiI140 sequences (OR124064-OR124621). Statistical analyses were conducted using GraphPad Prism version 7.00 for Mac (GraphPad Software, La Jolla, CA, USA). The generation of graphs was carried out using Excel (version 16.75.2) and Powerpoint (version 16.72, Microsoft, Redmond, WA, USA). Phylogenetic trees were produced using Geneious Pro 10.1.2 and PhyML 3.3.20180214 [23,24]. The analysis underwent bootstrap testing (100 replicates) with the optimal tree shown and drawn to scale. The evolutionary distances were computed using the maximum composite likelihood method [25] and diagrammed based on midpoint rooting.

## 3. Results

### 3.1. Characterization of the Mitochondrial sCOIB Segment

**Figure 1:**
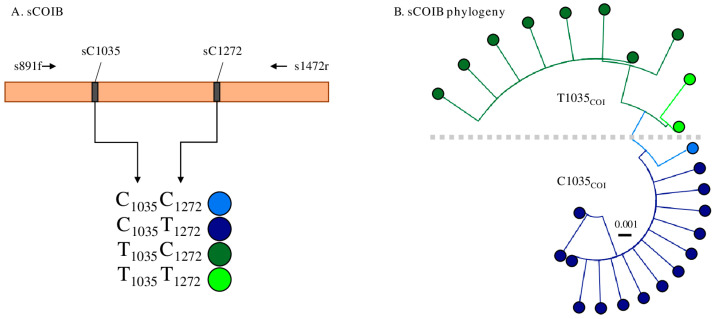
Description of the sCOIB SNPs and the T1035 and C1035 haplotypes. (**A**) Diagram of the sCOIB fragment with horizontal arrows indicating primers used for PCR amplification and DNA sequencing. The observed polymorphisms produced by the two most frequent polymorphic sites (sC1035 and sC1272) in the segments are listed. The pairings represent haplotypes and are color-coded for the rest of the figure. (**B**) Maximum-likelihood phylogeny of the sCOIB variants observed. Dashed line separates the T1035 and C1035 haplotypes.

A pair of mitochondrial haplotypes were previously shown to detect a genetic structure in a survey of SBL in the U.S. [26]. These were identified from the 3′ portion of the SBL mitochondrial *COI* gene, a fragment designated as sCOIB, from which nine SNPs were identified. Only two of the nine SNPs were polymorphic in more than one sample, sC1035 and sC1272, and these produced four pairwise combinations, with the C_1035_T_1272_ and T_1035_C_1272_ haplotype categories predominating (Figure 1A). Sequence analysis of 1405 specimens revealed 25 variants that via maximum-likelihood phylogeny analysis could be separated into two groups based solely on the sC1035 polymorphism (Figure 1B). These are subsequently referred to as either the T1035_COI_ or C1035_COI_ haplotype depending on the presence of T_1035_ or C_1035_, respectively.

### 3.2. Linkage between i65del_Tpi_ and T1035_COI_

**Figure 2:**
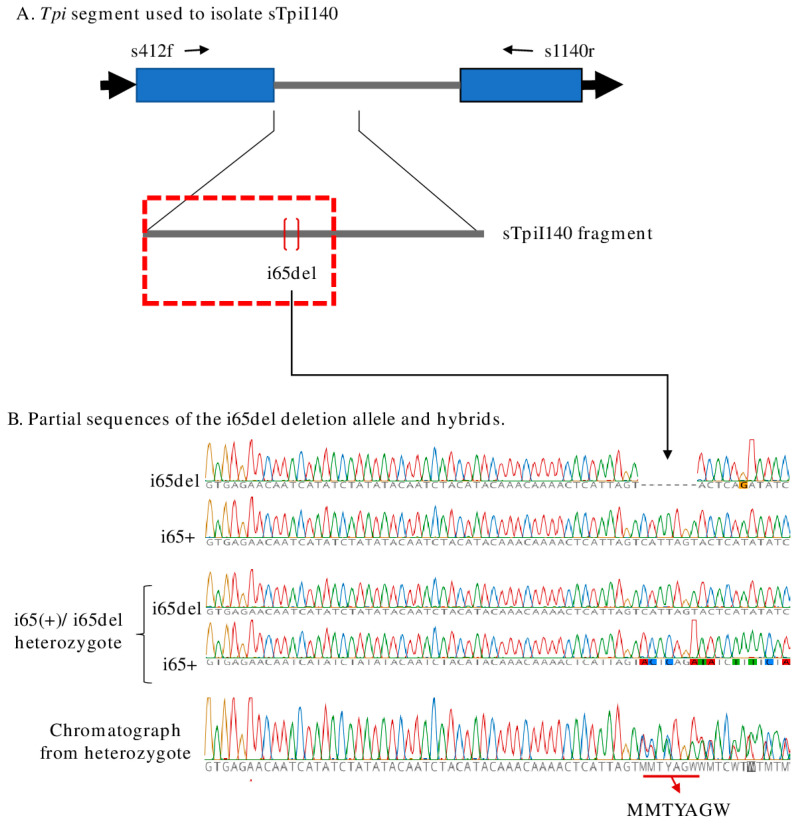
Description of the sTpiI140 segment from the SBL *Tpi* gene. (**A**) Map of portion of the SBL *Tpi* gene showing the two exons (blue boxes) separated by the intron containing the sTpiI140 fragment. One sTpiI140 variant contains a 7-bp deletion. Thin arrows depict the approximate location of primers used for the PCR amplification of sTpiI140. (**B**) Sequence of the sTpiI140 portion outlined by the red box that shows the consensus sequences associated with the i65del and i65+ alleles. In i65del/i65+ heterozygotes, DNA sequencing of the amplified *Tpi* segment using the s412f primer produces a frameshift in the chromatograph beginning at the start of the i65del deletion that tarts with the diagnostic MMTYAGW sequence.

A highly polymorphic intron segment from the Z-linked *Tpi* gene had previously been used to study the FAW host strains [17,20], and the analogous fragment from SBL, sTpiI140, was recently isolated and characterized (Figure 2A). A 7-bp deletion, designated as i65del_Tpi_, was found in the sTpiI40 segment that was present in 12% of the pooled collections (Figure 2A).

**Figure 3:**
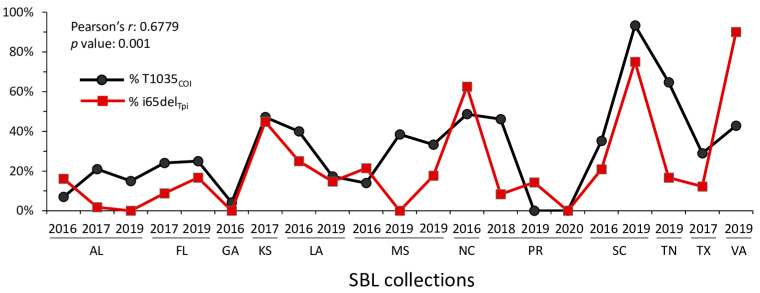
Frequencies of the T1035_COI_ mitochondrial haplotype and the i65del_Tpi_ allele in the SBL collections (Table 1). The test for correlation using Pearson’s *r* is indicated.

Comparisons of the distribution of the sCOIB mitochondrial haplotypes and sTpiI140 in our collections uncovered evidence of a possible linkage between i65del_Tpi_ and T1035_COI_. This was unexpected given that these markers reside on different genetic elements, the Z-chromosome for the former and the mitochondrial genome for the latter. This can be visualized via a direct comparison of the frequencies of T1035_COI_ and i65del_Tpi_ for each of the SBL collections (Figure 3). The similarity of the overlapping curves was confirmed via the test for correlation (Pearson’s *r* = 0.630; *p* = 0.002). The data indicate that the T1035_COI_ mitochondrial haplotype and i65del_Tpi_ allele segregate in combination at frequencies too high for what would be predicted from random mating.

Additional statistical support came from organizing the i65del_Tpi_ allele frequencies by state and comparing its frequencies in the C1035_COI_ and T1035_COI_ populations. Because the colony and field collections differed with respect to artificial rearing and source locations, the two data sets were analyzed separately as well as in combination. In each analysis, states with fewer than ten specimens of either the C1035_COI_ or T1035_COI_ haplotypes were pooled. Both the colony and field-collected specimens showed a significantly higher association of the i65del_Tpi_ allele with the T1035_COI_ mitochondrial haplotype (Table 2A,B).

Given the similarity in their results, the data from the colony and field collections were combined and the i65del_Tpi_ frequencies were then presented for each state, with the results for GA, VA, TN, and PR being pooled (Table 2C). Despite the considerable variability between states, the frequency of i65del_Tpi_ was consistently higher in the T1035_COI_ population, with the difference from the C1035_COI_ group ranging from a low one of 5% (MS) to as large as 32% (FL). As with the individual data set analyses, the observed difference between the means was statistically significant.

### 3.3. Frequency of i65del/+ Heterozygotes

A test for potential reproductive barriers was possible because the methodology used to identify i65del_Tpi_ also allowed the detection of heterozygotes between i65del_Tpi_ and i65+_Tpi_ (designated as i65del/+). The presence of both alleles in the same sequencing reaction will produce a chromatograph with a predictable superimposition of curves resulting from the frameshift beginning at the i65del breakpoint (Figure 2B). The overlapped region will begin with the diagnostic MMTYAGW sequence (M = T/A, Y = C/T, and W = A/T per the IUPAC convention).

The frequency of heterozygotes was used to calculate Wright’s local inbreeding coefficient, *F_is_*, which is a metric for quantifying observed heterozygosity (*H_o_*) relative to what would be expected (*H_e_*) from a population at the Hardy–Weinberg equilibrium, based on the equation *F_is_* = (*H_e_* − *H_o_*)/*H_e_*. A *F_is_* equal to 0 indicates a state of equilibrium where *H_e_* = *H_o_*, with values becoming increasingly positive if heterozygosity is suppressed (high inbreeding) and more negative if the observed heterozygosity is greater than expected. An added complication is that because *Tpi* is on the *Z*-chromosome, heterozygotes of two *Tpi* alleles can only occur in SBL males as females being *ZW* will only carry one copy of the gene. Our initial analysis was therefore limited to the pheromone trap collections that exclusively attracted adult males. The pooled data produced a *F_is_* of 0.30 while the mean *F_is_* of the six collections was 0.41 (Table 3A). Both highly positive values indicate the substantial suppression of hybrids. Only the Alabama collection (AL2017) gave a negative *F_is_* (−0.11).

The subsequent analysis of the remaining collections (limited to those with sample numbers of >20) gave similar results even though the gender of the specimens was not determined. The likely presence of female specimens reduces the opportunities for i65del/+ hybrids and therefore should lead to overestimates of *F_is_*. The results are consistent with this reasoning with both pooled and mean *F_is_* values being higher than that observed with the pheromone trap collections (Table 3B). In the GA2016 collection, the i65del_Tpi_ allele was not found either as a homozygote or heterozygote and so a calculation of *F_is_* could not be performed. Otherwise, the larva results support the findings from the pheromone trap data set as a consistently positive *F_is_* was observed that was indicative of suppressed heterozygosity.

### 3.4. The Phylogeny of the sTpiI140 Segment

**Figure 4:**
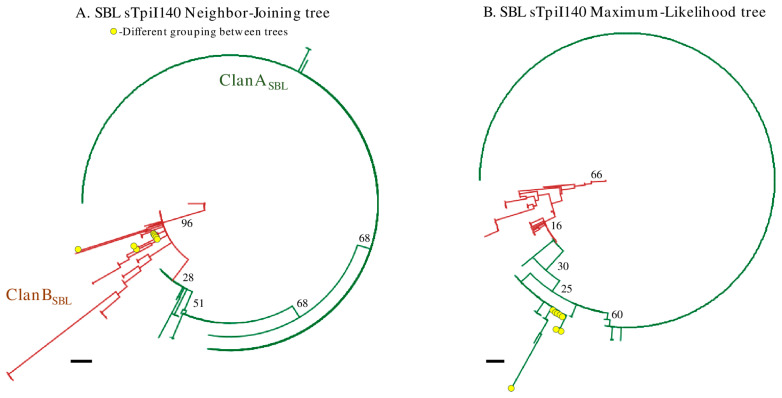
Phylogenetic analysis of the SBL sTpiI140 segment identifying taxonomic groups such as those found using the analogous fTpi100 segment from FAW. (**A**) Neighbor-joining phylogenetic tree produced from 550 SBL sTpiI140 sequences. The ClanA_SBL_ and ClanB_SBL_ groups are identified by color. Numbers indicate bootstrap frequency. (**B**) Maximum-likelihood treatment of same data set with colors indicating analogous groups. Ovals identify sequences found in the ClanB group in the neighbor-joining tree. Scale bar denotes a phylogenetic distance of 0.01 nucleotide substitutions per site.

Neighbor-joining phylogenetic analysis of the SBL sTpiI140 sequence from 550 specimens collected from multiple U.S. locations and times produced a tree that could be divided into two groups based on the distribution of i65del_Tpi_ allele (Figure 4A). The tree is unrooted because of the high variability of the intron segment that curtailed finding an appropriate outgroup. Therefore, the groups are designated as clans indicating a phylogenetic split but with the direction of evolution unspecified. We identified one group as ClanB_SBL_, which was made up of 66 individuals. The remaining sequences, designated as ClanA_SBL_, emerge from a single branch out of ClanB_SBL_, with 88% of the 484 sequences of a single haplotype. The i65del_Tpi_ allele was asymmetrically distributed between the two SBL groups, with 100% (66/66) carrying i65del_Tpi_ in ClanB_SBL_ compared to none out of 492 in ClanA_SBL_ (NJ, Table 4). Based on these observations, i65+_Tpi_ and i65del_Tpi_ are accurate molecular proxies for ClanA_SBL_ and ClanB_SBL_, respectively. The sCOIB haplotypes also showed a disproportional distribution, though not to the same degree as that of the *Tpi* alleles. The T1035_COI_ haplotype made up 41% of ClanB_SBL_, but only 17% of ClanA_SBL_. Haplotype diversity was more than four-fold higher in ClanB_SBL_ than ClanA_SBL_ (0.935 vs. 0.229) and nucleotide diversity was eight-fold greater (0.024 vs. 0.003).

Because the neighbor-joining tree was poorly supported by bootstrapping, the same dataset was analyzed via maximum-likelihood analysis (Figure 4B). The results were similar with respect to the identification of groupings with asymmetric distributions of the i65del_Tpi_ and T1035_COI_ markers (ML, Table 4). Eight specimens were grouped differently with the two methods resulting in only minor variations in marker frequencies and genetic diversity metrics. Bootstrap support was again weak, indicating that the details of the phylogenetic pattern are uncertain. However, both tree building methods identified two phylogenetic groups with similar differences in marker distributions, consistent with the identification of two distinct populations.

## 4. Discussion

The experimental strategy applied in this study on SBL was based on methods used to investigate the FAW host strains, with the results suggesting similarities in the population structure of the two species. One example came from comparisons of the two groups identified via the phylogenetic analysis of sTpiI140 (Figure 4) with that derived from a portion of the analogous *Tpi* intron in FAW that differentiates the C-strain and R-strain [20]. Like the SBL ClanA and ClanB, the phylogenetic groups associated with the FAW host strains differ in the frequencies of *Tpi* and *COI* markers as well as with haplotype diversity and nucleotide diversity [20]. The FAW strains are believed to diverge via a process that involves differences in host usage, and it is tempting to hypothesize that something similar may be happening with SBL, though we have no evidence yet of any population structure correlated with plant hosts or habitats. Testing for these will require systematic sampling from different plant hosts over multiple locations and times.

Additional evidence for two distinct SBL populations was derived from the fact that the nuclear Z-chromosome and the mitochondrial genome are separate genetic entities that segregate independently of the other. This means that under conditions of random mating, the i65del_Tpi_ allele should display no linkage with a mitochondrial haplotype and therefore should be found in equal proportions in the T1035_COI_ and C1035_COI_ populations. Our observations of linkage disequilibrium between these markers (Table 2) indicate nonrandom mating behavior that we believe is most simply explained by two populations with limited capacity for successful cross-hybridization, one disproportionately predominated by T1035_COI_ i65del_Tpi_ (ClanB_SBL_) and the other predominated by C1035_COI_ i65+_Tpi_ (ClanA_SBL_).

This suggestion is supported by our ability to identify i65del/+ heterozygotes, which allowed the quantification of the hypothesized reproductive restrictions between the i65del_Tpi_ and i65+_Tpi_ populations. The *F_is_* calculations showed consistent evidence of high levels of inbreeding diagnostic of the Wahlund effect that describes a reduction in observed heterozygosity as populations diverge into multiple groups [27] (Table 3). The reduced frequency of cross-hybridization between i65del_Tpi_ and i65+_Tpi_ populations as indicated by the *F_is_* data provides compelling explanation for the asymmetric distribution of the T1035_COI_ and C1035_COI_ mitochondrial haplotypes across these groups.

Given the similarities between FAW and SBL with respect to their overlapping host range, geographical distribution, and climate suitability, it is plausible that SBL is facing similar selective pressure to diverge into two populations. This can be difficult to demonstrate, however, as populations at intermediate stages of speciation are, by definition, still capable of some cross-hybridization with potentially high levels of gene flow. In addition, the genes driving divergence are likely to be low in number early in the speciation process and many of the polymorphisms that become asymmetrically distributed by chance will likely vary by region. Given these issues, our phylogenetic identification of ClanA_SBL_ and ClanB_SBL_ that seem by analogy to FAW to be good candidates for diverging populations represents an important advance in understanding SBL population structure.

In summary, evidence was presented based on the distribution of *COI* and *Tpi* polymorphisms for two sympatric SBL populations with only a limited capability for cross-hybridization. These findings complement results from a companion paper demonstrating geographical differences in haplotype distribution that may reflect how SBL migration is impacted by the timing of soybean agriculture. Together, they describe a more complicated population biology in the United States than previously thought with potential relevance to the dispersion of resistance traits and projections of SBL migration.

## Figures and Tables

**Table 1:** Source information for SBL collections. All collections from soybean.

Collection	State	Year	Type	Collector
A. Field collections				
AL2017	Alabama	2017	Pheromone trap	R. Meagher
FL2017	Florida	2017	Pheromone trap	R. Meagher
FL2019	Florida	2019	Pheromone trap	R. Meagher
MS2019a	Mississippi	2019	Pheromone trap	R. Meagher
TX2017	Texas	2017	Pheromone trap	R. Parker
KS2017	Kansas	2017	Pheromone trap	B. McCornack
PR2018	Puerto Rico	2018	Larvae, field	H. Portillo
PR2019	Puerto Rico	2019	Larvae, field	H. Teran
B. Colony collections				
AL2016	Alabama	2016	F1 generation	J. Davis
GA2016	Georgia	2016	F1 generation	J. Davis
LA2016	Louisiana	2016	F1 generation	J. Davis
MS2016	Mississippi	2016	F1 generation	J. Davis
NC2016	N. Carolina	2016	F1 generation	J. Davis
SC2016	S. Carolina	2016	F1 generation	J. Davis
AL2019	Alabama	2019	F1-2 generation	F. Musser, B. Catchot
LA2019	Louisiana	2019	F1-2 generation	F. Musser, B. Catchot
MS2019b	Mississippi	2019	F1-2 generation	F. Musser, B. Catchot
PR2020	Puerto Rico	2020	F1-2 generation	F. Musser, B. Catchot
SC2019	S. Carolina	2019	F1-2 generation	F. Musser, B. Catchot
TN2019	Tennessee	2019	F1-2 generation	F. Musser, B. Catchot
VA2019	Virginia	2019	F1-2 generation	F. Musser, B. Catchot

**Table 2:** Comparisons of i65del_Tpi_ allele frequencies in the two sCOIB haplotype populations in the colony (A), field (B), and combined collections (C). Frequencies are in percentages, “n” refers to sample numbers, and an asterisk indicates that the observed difference between the mean frequencies is statistically significant.

		i65del_Tpi_ Frequency (%)		*t*-Test Scores
Collection	n	C1035_COI_	T1035_COI_	*p* (*t, df*)
A. Mean Colony ^1^	410	18 ± 5	47 ± 11	*p* = 0.007 * (5.203, 4)
B. Mean Field ^2^	533	18 ± 5	27 ± 11	*p* = 0.037 * (3.075, 4)
C. Colony + Field				
AL	144	16	26	
FL	190	14	46	
KS	53	27	42	
LA	92	13	54	
MS	80	24	29	
NC	37	21	44	
SC	86	14	36	
TX	145	20	42	
GA-VA-TN-PR	116	13	48	
Mean ± sd		18 ± 5	41 ± 9	*p* = 0.004 * (5.808, 8)

^1^ LA2016, LA2019, MS2016, MS2019b, NC2016, SC2016, SC2019, and Pool (AL2016, AL2019, GA2016, PR2020, TN2019, and VA2019). ^2^ AL2017, FL2017, FL2019, KS2017, TX2017, and Pool (MS2016, MS2019a, PR2018, and PR2019). *, significant difference.

**Table 3:** Comparisons of heterozygosity. Numbers of relevant genotypes and calculations of Wright’s inbreeding coefficient, *F_is_*, for all collections with sample sizes greater than 20.

A. Male adults	Genotype (number)			
Collection	i65del/i65del	+/+	i65del/+	*F_is_*
AL2017	1	56	25	−0.11
FL2017	11	125	26	0.36
FL2019	4	24	0	1.00
KS2017	13	29	11	0.54
MS2019a	3	17	4	0.49
TX2017	11	90	44	0.14
Total	43	341	109	0.30
			Mean	0.41
B. Larvae of unknown gender				
Collection	i65del/i65del or i65del/W	+/+ or +/W	i65del/+	*F_is_*
AL2016	5	31	7	0.49
GA2016	0	24	0	nd ^1^
LA2016	7	28	5	0.65
LA2019	5	34	13	0.27
MS2016	6	28	9	0.43
NC2016	10	16	11	0.39
PR2019	3	21	3	0.60
SC2016	9	43	19	0.31
VA2019	9	10	2	0.81
Total	54	235	69	0.48
			Mean	0.49

^1^ No i65del_Tpi_ alleles were detected so *F_is_* could not be calculated.

**Table 4:** Metrics from neighbor-joining (N-J) and maximum-likelihood (M-L) phylogenetic analyses of the sTpiI140 intron segment (sd = standard deviation).

Metric	Tree Type	ClanA	ClanB
*Tpi* marker: i65del_Tpi_	NJ	0%	100%
	ML	2%	98%
*COI* marker: T1035_COI_	NJ	17%	41%
	ML	18%	41%
Haplotype diversity (±sd)	NJ	0.229 ± 0.025	0.935 ± 0.014
	ML	0.194 ± 0.024	0.943 ± 0.011
Nucleotide diversity (±sd)	NJ	0.003 ± 0.000	0.024 ± 0.144
	ML	0.002 ± 0.001	0.026 ± 0.001

## Data Availability

Relevant DNA sequence data are openly available in Genbank. All other data are present in this study.
